# Older Adults Walking the Camino de Santiago Pilgrimage: Motivations, Transformations, and Spiritual Perspectives

**DOI:** 10.1093/geroni/igaa057.2357

**Published:** 2020-12-16

**Authors:** Holly Nelson-Becker, Joe Pickard, Gina Aitch, Alyssa Buettner

**Affiliations:** 1 Brunel University London, Uxbridge, England, United Kingdom; 2 University of Missouri-St. Louis, St. Louis, Missouri, United States; 3 University of Missouri-St. Louis, st. louis, Missouri, United States

## Abstract

This mixed-method study describes reasons that older people chose to complete the Camino de Santiago pilgrimage route in Spain and their assessment of how they were changed by the experience. The study is framed in Maslow’s (1988) self-actualization and Tornstam’s (2005) concept of gerotranscendence. We analyzed a subset of 121 participants age 65 and over who completed an online survey. Motivation included five themes: gratitude and transitions, cultural or historical curiosity. relationships, challenge and adventure, and spirituality. Transformations since their return involved greater strength, self-understanding, peace, desire to live lightly and to integrate their experience. Older individuals who walked the Camino have done so for a variety of reasons. Spiritual reasons may be more difficult to disclose. Half responded in the open-ended question, but a later spirituality question added many more respondents. Older people envision many forms of benefit to walking the pilgrimage and find growth in the experience.

